# A Rare Incidental Finding: Heterotopic Salivary Gland Tissue Adjacent to Dermatofibrosarcoma Protuberans of the Abdominal Wall

**DOI:** 10.1155/crip/6514245

**Published:** 2026-05-20

**Authors:** Leticia Boeta Ángeles, Diana Karen Brito Bustillos, Jorge Alberto Cortez Vila, Tania Raisha Torres Victoria, María Elisa Vega Memije, Rosa María Lacy Niebla

**Affiliations:** ^1^ Department of Dermatology, Hospital Juárez del Centro, Mexico City, Mexico; ^2^ Department of Dermatopathology, Hospital General Dr. Manuel Gea González, Mexico City, Mexico, hospitalgea.salud.gob.mx; ^3^ Division of Dermatology, Hospital General Dr. Manuel Gea González, Mexico City, Mexico, hospitalgea.salud.gob.mx; ^4^ Private Dermatology Practice, Mexicali, Mexico

**Keywords:** dermatofibrosarcoma protuberans, heterotopic salivary gland tissue, Mohs micrographic surgery

## Abstract

**Summary:** This case presents incidental heterotopic salivary gland tissue adjacent to a dermatofibrosarcoma protuberans (DFSP) located in the abdominal wall. The mature, functional heterotopic tissue was identified during Mohs micrographic surgery (MMS) performed for a DFSP, a slow‐growing cutaneous tumor with locally aggressive and potentially malignant behavior. Definitive diagnosis of the heterotopic tissue was confirmed by histopathological examination and immunohistochemical analysis. This case underscores the importance of being alert to unusual tissue findings at the time of MMS and the value of close collaboration between dermatologists and pathologists.

## 1. Introduction

Dermatofibrosarcoma protuberans (DFSP) is a rare fibrohistiocytic sarcoma of the skin characterized by intermediate malignant potential. It originates from dermal fibroblasts and most commonly arises on the trunk, followed by the lower extremities, head and neck, and less frequently the upper extremities. Clinically, it starts as a slow‐growing infiltrative plaque and progresses to nodules or tumors that can ulcerate or bleed, and it rarely metastasizes [1–3].

Although DFSP has a low incidence of metastasis and a low mortality rate, it is prone to local recurrence due to its deceptive and insidious tumor extensions, especially in depth. Therefore, complete surgical excision with negative histopathological markers is crucial for successful treatment. Mohs micrographic surgery (MMS) has become an ideal treatment option for DFSP as it allows immediate examination of all cut surgical margins during the procedure [1, 4, 5].

Any skin tumor may merely be associated with other pathological processes, posing a diagnostic challenge. A complete histological examination of the DFSP in this case revealed heterotopic salivary gland tissue. We present a case of DFSP with adjacent heterotopic salivary gland tissue.

## 2. Clinical Case

**Figure 1:**
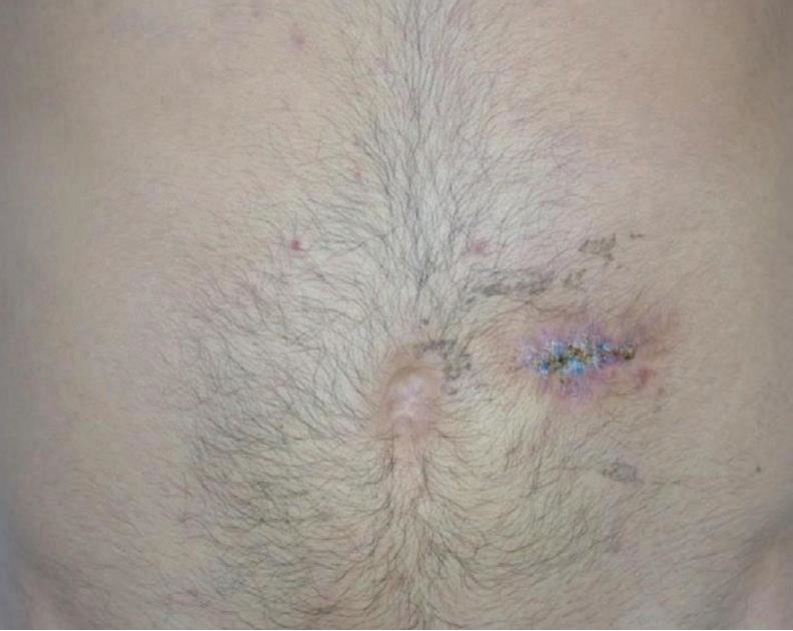
Clinical appearance of dermatofibrosarcoma protuberans of the mid left abdominal wall. Sutures present from a recent incisional biopsy.

A 29‐year‐old man presented with a 20‐year history of a slowly evolving lesion on the midabdomen and left flank. At the time of physical examination, a poorly circumscribed 45 × 60 − mm subcutaneous mass was found, not fixed to underlying structures, with a recent linear biopsy surgical scar with sutures at its center (Figure 1). The lesion initially appeared as an asymptomatic, approximately 5‐mm violaceous papule and progressively enlarged over the preceding 2 years, without response to topical or intralesional corticosteroids. During this time, the lesion developed localized alopecia and mild pruritus. The patient underwent surgical excision by a plastic surgeon; however, postoperative wound dehiscence and progressive tumor enlargement prompted referral to our dermatology service.

**Figure 2:**
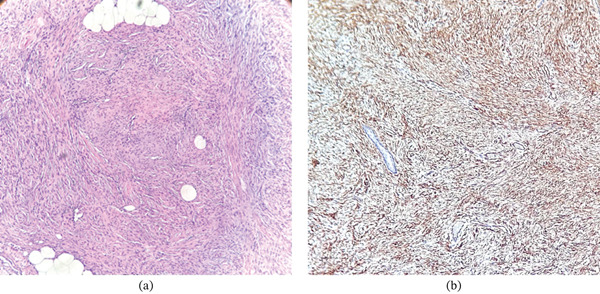
Histopathologic appearance of dermatofibrosarcoma protuberans. (a) Hematoxylin eosin staining shows spindle cells arranged in a storiform pattern surrounding adipocytes, producing the characteristic honeycomb pattern (original magnification ×10). (b) CD34 immunohistochemical staining demonstrates diffuse membranous positivity in the neoplastic spindle cells (original magnification ×10).

Histopathologic examination of an incisional biopsy (Figure 2a) demonstrated a dermal‐based spindle cell proliferation composed of slender cells with elongated nuclei, arranged in a storiform pattern with focal parallel alignment, infiltrating the subcutaneous tissue. Immunohistochemical staining revealed diffuse CD34 positivity within the spindle cell population, as well as in associated vascular structures (Figure 2b), supporting the diagnosis of DFSP. Magnetic resonance imaging revealed a solid, heterogeneous mass within the anterior abdominal wall at the left mesogastric region, adjacent to the umbilical scar. The lesion was iso‐ to hyperintense relative to skeletal muscle, with irregular margins, involving the dermis, subcutaneous tissue, and fascia of the left rectus abdominis muscle. It measured approximately 5.6 × 3.5 cm in greatest dimensions with a maximal thickness of 1.2 cm and demonstrated avid enhancement following gadolinium administration.

MMS was performed in three stages until tumor‐free margins were achieved, resulting in a final defect measuring 9.5 × 10.5 cm, with margins of 5 and 6 cm at either end and extension to the deep muscle fascia. Reconstruction was subsequently performed by the plastic surgery team.

**Figure 3:**
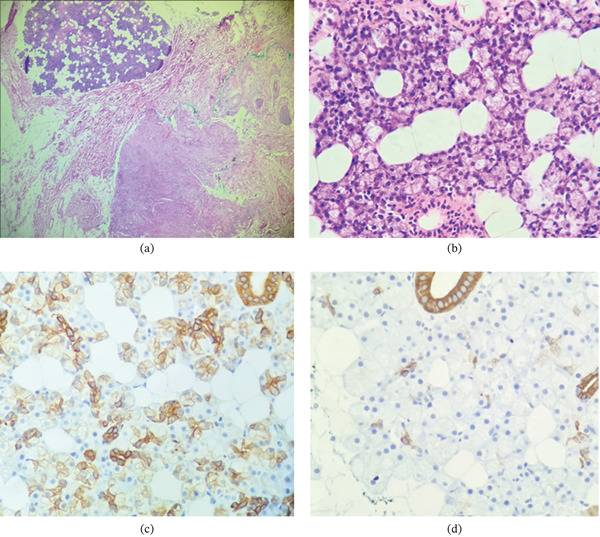
Ectopic salivary gland tissue adjacent to dermatofibrosarcoma protuberans. (a) Low‐power hematoxylin–eosin view shows lobules of glandular tissue within fibrous septa, adjacent to dermatofibrosarcoma protuberans (lower right) (original magnification ×4). (b) High‐power view shows glandular epithelium with interspersed adipocytes and scattered excretory ducts (original magnification ×40). (c) Cytokeratin 7 immunohistochemical staining shows positivity in glandular cells and ducts (original magnification ×40). (d) Cytokeratin 19 immunohistochemical staining shows positivity in ducts and negativity in glandular cells (original magnification ×40).

Histopathological examination of tissue from the first and second MMS stages revealed, in addition to DFSP, a distinct cellular proliferation within the deep reticular dermis and hypodermis adjacent to the DFSP. This component was characterized by glandular acini arranged in lobules separated by fibrous septa (Figure 3a), composed of two cell populations—serous and mucinous. Scattered ducts lined by cuboidal cells and occasional mature adipocytes were also identified between the glands (Figure 3b). Morphologically, this tissue was consistent with mixed‐type salivary gland tissue.

Immunohistochemical studies demonstrated positivity of the glandular acini for Cytokeratin 7 (Figure 3c), whereas the ducts were positive for Cytokeratins 7 and 19 (Figure 3d). Based on the histopathological features and heterotopic location, a diagnosis of salivary gland choristoma associated with DFSP was established.

## 3. Discussion

The presence of mature, well‐differentiated, and organized noncancerous tissue in an abnormal anatomic location is said to be heterotopic. When this tissue forms a distinct clinical mass, it is known as choristoma [6].

These anomalous tissues have been identified in various anatomic sites, including cervical lymph nodes, the mastoid process, external auditory canal, tongue, sternoclavicular joint, and thyroid and parathyroid glands [7]. However, no occurrences involving the abdominal wall have been documented thus far.

The presence of salivary gland choristomas in these locations supports the theory that they arise from the persistence of aberrant vestigial tissues or from defects in the closure of the precervical sinus within the branchial apparatus [8]. However, the abdominal wall location seen in this case supports a different etiopathogenic mechanism. Salivary gland choristomas have been described in the rectal mucosa, supporting the hypothesis that during early embryogenesis, pluripotent endodermal stem cells may evade primary induction and migrate to ectopic sites, where local stimuli or microenvironmental factors may promote differentiation [9]. In this context, the associated DFSP may have contributed to the differentiation of the ectopic salivary gland tissue observed in this case or it may be a coincidental finding.

About 80% of salivary gland choristomas are benign in terms of biological behavior, whereas the remaining cases may develop into neoplasms such as acinic cell carcinomas, mucoepidermoid carcinomas, pleomorphic adenomas, Warthin tumors, and adenocarcinomas. Clinically, these lesions may present in childhood as nodular, cystic, or fistulized lesions with saliva‐like discharge [10]. In the present case, the patient did not report symptoms attributable to the heterotopic tissue.

Most choristomas are discovered incidentally or in association with secondary neoplasms [8], as observed in the present case. To date, no cases of heterotopic tissues or choristomas associated with DFSP have been reported. However, Globerson [11] described a salivary gland heterotopic tissue associated with basal cell carcinoma of the neck, identified during MMS.

The lesion identified during MMS in our patient posed a diagnostic challenge, particularly for clinicians unfamiliar with incidental heterotopic tissue, specifically salivary gland. Although it was clear that the tissue did not belong to DFSP, it remained unclear whether it was a salivary or pancreatic heterotopic tissue. This differentiation was particularly important given the abdominal position, because pancreatic choristomas of the abdominal wall, while uncommon, have been recorded. Thus, immunohistochemistry tests were required to confirm the morphologic impression [12, 13]. Careful examination of such materials is required, as up to 20% may include malignant components or be part of the clinical spectrum of metastatic disease [8, 14].

After a comprehensive review of the medical literature, we found no reports of incidental heterotopic tissue associated with a DFSP.

## 4. Conclusion

Accurate diagnosis and distinction between benign and malignant soft tissue lesions are essential to patient care. In the setting of salivary gland choristomas, the diagnostic challenge is heightened, making confirmation with immunohistochemical studies essential. This case highlights the importance of precise histopathologic identification, as heterotopic tissue may obscure diagnosis, particularly when encountered incidentally during MMS. Familiarity with extracutaneous pathology and close collaboration between dermatologists and pathologists are critical for the appropriate recognition and management of these unusual associations.

## Funding

No funding was received for this manuscript.

## Conflicts of Interest

The authors declare no conflicts of interest.

## Data Availability

Data sharing not applicable to this article as no datasets were generated or analyzed during the current study.
